# Conservation physiology across scales: insights from the marine realm

**DOI:** 10.1093/conphys/cou024

**Published:** 2014-07-08

**Authors:** Steven J. Cooke, Shaun S. Killen, Julian D. Metcalfe, David J. McKenzie, David Mouillot, Christian Jørgensen, Myron A. Peck

**Affiliations:** 1 Fish Ecology and Conservation Physiology Laboratory, Department of Biology and Institute of Environmental Science, Carleton University, 1125 Colonel By Drive, Ottawa, ON, Canada K1S 5B6; 2 Institute of Biodiversity, Animal Health, and Comparative Medicine, Graham Kerr Building, University of Glasgow, Glasgow G12 8QQ, UK; 3 Centre for Environment, Fisheries and Aquaculture Science (Cefas), Lowestoft Laboratory, Suffolk NR33 0HT, UK; 4 Equipe Diversité et Ecologie des Poissons, UMR5119 Ecologie des Systèmes Marins Côtiers, Université Montpellier 2, Place Eugène Bataillon, 34095 Montpellier cedex 5, France; 5 Uni Computing, Uni Research, PO Box 7810, NO-5020 Bergen, Norway; 6 Institute of Hydrobiology and Fisheries Science, University of Hamburg, Olbersweg 24, D-22767 Hamburg, Germany

**Keywords:** Body size, downscaling, marine, scale, upscaling

## Abstract

As the field of conservation physiology develops and becomes increasingly integrated with ecology and conservation science, the fundamental concept of scale is being recognized as important, particularly for ensuring that physiological knowledge is contextualized in a manner most relevant to policy makers, conservation practitioners and stakeholders. Failure to consider the importance of scale in conservation physiology—both the challenges and the opportunities that it creates—will impede the ability of this discipline to generate the scientific understanding needed to contribute to meaningful conservation outcomes. Here, we have focused on five aspects of scale: biological, spatial, temporal, allometric and phylogenetic. We also considered the scale of policy and policy application relevant to those five types of scale as well as the merits of upscaling and downscaling to explore and address conservation problems. Although relevant to all systems (e.g. freshwater, terrestrial) we have used examples from the marine realm, with a particular emphasis on fishes, given the fact that there is existing discourse regarding scale and its relevance for marine conservation and management. Our synthesis revealed that all five aspects of scale are relevant to conservation physiology, with many aspects inherently linked. It is apparent that there are both opportunities and challenges afforded by working across scales but, to understand mechanisms underlying conservation problems, it is essential to consider scale of all sorts and to work across scales to the greatest extent possible. Moreover, given that the scales in biological processes will often not match policy and management scales, conservation physiology needs to show how it is relevant to aspects at different policy/management scales, change the scales at which policy/management intervention is applied or be prepared to be ignored.

## Abstract (precis)

The concept of “scale” (including biological, spatial, temporal, allometric and phylogenetic aspects) is fundamental to conservation physiology. Failure to consider its importance will impede our ability to contribute to meaningful conservation outcomes. It is essential to consider scale of all sorts and to work across scales to the extent possible.

## Context

Scale is a fundamental concept in ecology (Levin, 1992; Peterson and Parker, 1998; Schneider, 2001). The problem of scale and scaling arises because the scale at which we can measure something is only rarely the same scale at which we are interested in its consequences. Depending on context and the types of processes involved, scaling can be defined in many different ways (see Schneider, 2001). In each of the following examples, a different type of scaling is involved: we may measure the location of some individuals when we are really interested in the distribution of the entire population; we may measure bioenergetics of some life stages in the laboratory when we are really interested in the lifetime consequences for free-living individuals; we may measure a physiological rate in cells or tissues when we are really interested about the performance of the organ or the organism; or we may measure the effects of decreased pH and increased partial pressure of CO_2_ associated with ocean acidification during short-term experiments lasting days to weeks on a few key species when we are really interested in the longer-term (decadal) consequences to the functioning of a marine ecosystem. In each case, we would need to rely on some method for scaling from the level of observation to the level at which we make inferences.

The concept of scale is also highly relevant to conservation science and resource management (Noss, 1992). The concept of biological hierarchies or levels of organization (e.g. genes, cells, organs, populations; West and Brown, 2005) is a key element of conservation physiology and pivotal for understanding how effects on the physiology of the individual translate into impacts on populations, communities and ecosystems. One must also decide at what temporal and spatial scale one is going to monitor biodiversity (Bunnell and Huggard, 1999; Poiani *et al.*, 2000), assess extinction risk (Hartley and Kunin, 2003), generate and implement conservation strategies (Pressey and Bottrill, 2009), take restorative action (Lewis *et al.*, 1996; Poiani *et al.*, 2000; Bosch *et al.*, 2004) and, finally, prioritize conservation efforts in a context of limited resources (Hartley and Kunin, 2003). Most conservation issues further require the integration of insights and studies at different scales to ensure that a species or population is self sustaining over time.

As the field of conservation physiology (see Wikelski and Cooke, 2006; Cooke *et al.*, 2013a) develops and becomes integrated with ecology and its application (e.g. conservation science and resource management), the concept of scale is increasingly being regarded as important, particularly for ensuring that physiological knowledge is contextualized in a manner most relevant to policy makers, resource managers, conservation practitioners and stakeholders (Cooke and O'Connor, 2010). Beyond biological, temporal and spatial scales, allometric scaling of biological processes (such as metabolism and growth) with body size (e.g. Schmidt-Nielsen, 1984, 2005; West *et al.*, 1997) is also relevant. Some have suggested that phylogenetic scale is also relevant for conservation physiology because variation in physiological traits is not independent of phylogenetic relationships among species, which integrate long-term evolutionary processes (Chown and Gaston, 2008). Given that there are essentially five types of biological scales relevant to conservation physiology, it seems appropriate to think about how conservation physiology works across scales. However, extending beyond biological scales, but highly relevant to conservation physiology, is the scale of policy development, decision support (e.g. models), management actions and stakeholder behaviour. Bearing in mind that biological processes of interest will often not match policy (including political elements) and management scales, science either needs to show how it is relevant to aspects at the policy/management scale, change the scale at which policy/management intervention is applied or prepare to be ignored.

Based on this background, we submit that failure to consider the importance of scale in conservation physiology—both the challenges and opportunities that it creates—will impede the ability of this discipline to generate the scientific understanding needed to contribute to meaningful conservation outcomes. To that end, the purpose of this article is to consider the challenges and opportunities for conducting and operationalizing conservation physiology research across scales. We focus on five aspects of scale, namely biological, spatial, temporal, allometric and phylogenetic. We also consider the scale of policy and policy application relevant to the five types of scale as well as the merits of upscaling and downscaling to explore and address conservation problems. We focus examples on the marine realm, with a particular emphasis on fishes, given the fact that there is existing discourse regarding scale and its relevance for marine conservation and management (e.g. Young *et al.*, 2006; Helmuth, 2009). Moreover, there is an active conservation physiology community engaged in marine systems as codified by the European Union Cooperation in Science and Technology (COST) Action on the Conservation Physiology of Marine Fishes (see http://www.cost.eu/domains_actions/fa/Actions/FA1004), to which all authors belong. Nonetheless, we submit that the concepts and ideas discussed here are equally relevant to aspects of conservation physiology and research in other systems (e.g. inland waters, terrestrial ecosystems).

## Biological scale

Biological scale refers to the hierarchical nature of biological organization, from genes, through organisms, populations and communities, all the way to ecosystems. Biological scale is, therefore, at the heart of conservation physiology as a discipline (Le Quesne and Pinnegar, 2012; Seebacher and Franklin, 2012). Considering the marine realm, the impacts of environmental change, whether local (habitat modifications, pollution, hypoxic events) or global (ocean warming and acidification), are exerted at the molecular and cellular level, influencing biochemical and physiological processes, as well as gene expression. These effects influence organ function that, in turn, translates into impacts on the metabolism and performance of the whole organism in its habitat. This then has effects on fitness-related functions, such as growth and reproduction, but can also drive behaviours such as habitat selection or migratory patterns. As a result, physiological effects can translate into effects on population dynamics and patterns of abundance. It has been suggested that individual physiology is, therefore, a ‘filter’ between environmental conditions and population-level impacts (Seebacher and Franklin, 2012). Such impacts on populations then translate into changes at an ecosystem scale, influencing the composition and biodiversity of communities and assemblages. It is at these higher organizational levels that societal impacts occur, which require management and policy decisions (Metcalfe *et al.*, 2012).

The prevailing wisdom is that marine ectotherms, such as fishes, have adapted over evolutionary time to a specific set of environmental conditions within which, on average, they function optimally (Claireaux and Lefrançois, 2007; Pörtner and Knust, 2007). Understanding the tolerance limits to environmental factors and the physiological mechanisms that determine them has potential to provide valuable insights into both current and future patterns of distribution and abundance of marine organisms (Pörtner and Farrell, 2008; Pörtner *et al.*, 2010). Physiological information can provide a mechanistic understanding of drivers of seasonal migratory behaviours (Cucco *et al.*, 2012) and of ongoing range shifts (Milazzo *et al.*, 2012) and, perhaps, inform invasive species control (Chown and Gaston, 2008). Measures of physiological performance or of underlying stress may also have potential as biomarkers of population productivity, vulnerability and resilience (Gagliano *et al.*, 2007; Cooke and O'Connor, 2010).

One major challenge is to understand how best to exploit individual physiological information to improve the predictive power of models of distribution and abundance. The current focus is on paradigms of energy flux (Jørgensen *et al.*, 2012), which is relevant to a variety of biological levels (e.g. communities, ecosystems; Tomlinson *et al.*, 2014). For ectotherms, such as fishes, it has been suggested that aerobic metabolic scope, defined as the difference between maximal aerobic metabolism and standard metabolism, can be used as a proxy for fitness, the so-called Fry paradigm (Fry, 1947, 1971; Ware, 1982; Kerr, 1990). Scope can be measured and modelled in relationship to environmental factors, such as temperature, and can then be used to predict an individual's physiological ‘power’ (Ware, 1982) as a function of prevailing or predicted conditions in their habitat (Claireaux and Lefrançois, 2007; Pörtner and Knust, 2007). In turn, one can make assumptions for the relationship between aerobic performance and fitness (e.g. Pörtner, 2010) or use more detailed models to assess the effect of bioenergetics on survival and reproduction and thus quantify fitness more explicitly (Jørgensen and Holt, 2013). This paradigm may be widely applicable to improve forecast models; for example, from the invasive potential of single species to the impacts of climate-change scenarios on community composition and biodiversity (Jørgensen *et al.*, 2012). The Dynamic Energy Budget model also offers a promising paradigm for modelling energy fluxes and potential for growth as a function of prevailing conditions (Nisbet *et al.*, 2012), as well as to inform interpretation of current and future patterns of distribution and abundance (Teal *et al.*, 2012).

A specific challenge is the slow-throughput nature of the physiological studies needed to parameterize models, especially for ectotherms, such as fishes, that display seasonal patterns of, for example, temperature acclimatization. A second challenge is how to incorporate intraspecific variation, such as patterns of local adaptation across a species' range, into models. Patterns of local adaptation across populations, as well as standing variation among individuals within a population, will be major factors defining the resilience of a population or species to environmental challenges and change (Frank and Brickman, 2001; Hughes *et al.*, 2005). Describing such patterns can also increase our mechanistic understanding of range shifts. Finally, biomarkers at one level of biological organization [e.g. endocrine (Sheriff *et al.*, 2011); oxidative stress indicator (Beaulieu *et al.*, 2013)] can be used to indicate population health, but such relationships need to be validated. Demonstrating that physiological information can improve the predictive power of model projections and provide valid biomarkers of the health of wild populations is an essential step for making physiology relevant to marine fisheries resource managers and policy makers.

## Temporal scale

Temporal scale refers to time, which can be crudely thought of as a continuum extending over the past, present and future. Temporal scales can range from the milliseconds of biochemical reactions to the eons that demark the chapters of Earth's geophysical and evolutionary history, and the significance of a specific scale can vary relative to the level of biological organization being considered and the type of conservation problem, as well as its spatial extent (see Szabo and Hedl, 2011; and below). For example, some biochemical processes relevant to organismal function associated with an acute stressor may occur on the scale of minutes to hours, while processes of relevance to populations may manifest themselves across generations if the stressor were to be chronic.

Temporal scale is of great relevance to most aspects of conservation science (Meine, 1999; Szabo, 2010), including conservation physiology (Cooke and O'Connor, 2010). In the design of experiments to characterize physiological responses and recovery dynamics of fish following stress associated with fisheries interactions (Cooke *et al.*, 2013b), the design of ecologically relevant climate-change scenario studies (Angilletta, 2009) or the prediction of effects of environmental change on different levels of biological organization (Seebacher and Franklin, 2012), temporal scale is a central concept. Helmuth (2009) noted that both temporal variability and history play a key role in driving the physiological responses of marine organisms to environmental change. In the context of thermal stress, the time scale of the stressor [e.g. acute thermal change event, such as a cold shock (Donaldson *et al.*, 2008) vs. sustained gradual warming associated with climate change (Roessig *et al.*, 2004)] will dictate to a large extent the capacity for organisms to acclimatize or for populations to evolve (Pörtner and Farrell, 2008; Helmuth, 2009). Not surprisingly, there is much conservation physiology research underway to understand both short- and long-term consequences of ocean acidification and warming ocean temperatures on marine fish and ecosystems. Conservation physiology also has much potential for understanding the mechanistic basis for the timing of key events, such as reproduction, and predicting how changes in environmental conditions (e.g. temperature, food quantity and quality) influence timing (Sims *et al.*, 2004; Lowerre-Barbieri *et al.*, 2011).

The concept of temporal scale creates a number of inherent challenges for conservation physiology. At the most simple level, it requires thinking about the evolutionary history of populations, the acclimatization history of individuals and the immense variation in time scale of relevance to biological processes. Experimental designs and interpretation of findings must be done with a view from proximate mechanisms to longer-term history. It is also necessary to consider long-term consequences (epigenetic effects and genetic adaptation between generations) and responses within a life-span (plasticity via acclimatization), given that some organisms can compensate for environmental changes (Seebacher and Franklin, 2012). Species with long generation times (e.g. decades) are predicted to have poor ability to respond to environmental stress (e.g. climate change) even on ecologically relevant time scales (Reusch and Wood, 2007), although, in many ways, that is only a hypothesis given the challenges in studying species with those life-history characteristics. The variation in biotic responses observed among individuals, populations and species reflects both past (generations) and recent experiences (e.g. days, hours), which are often quite variable among wild organisms. Given that conservation physiology tends to focus efforts on studying wild organisms (Wikelski and Cooke, 2006), it should be expected that the level of variation in responses to any treatments will be greater than if using cultured animals in a laboratory environment, which therefore may necessitate larger sample sizes, deliberate environmental perturbations (e.g. amplitude and/or frequency modulation of treatment levels) and different analytical approaches (Costa and Sinervo, 2004). Another challenge is reconciling time scales over which physiological studies can be conducted and interpreted with the longer-term context of an organism and its health, condition and fitness (i.e. ecological context).

## Spatial scale

Spatial scale refers to distance in space, ranging from nanometres for molecular structures in cells, centimetres or metres for the movements of small fish on reefs, to thousands of kilometres for the spawning migrations of open-ocean species, such as tuna, salmon and eels. For fishes in the marine environment, space is three dimensional, and they can make both vertical and horizontal movements. The spatial movements of marine fishes are largely driven by two factors, the spatial variability of marine habitats (Caddy, 2007) and the changing habitat requirements of a particular species (Gillanders *et al.*, 2003). Also, variation in light is an exogenous cue triggering huge diurnal and seasonal vertical migrations. Indeed, habitat variability will often have a component of temporal, usually seasonal, variability too, thereby linking spatial scale to temporal scale. Marine habitats are defined by a number of physical variables, such as depth, water currents, topography, sediment type, temperature, salinity, oxygen and illumination, plus biotic variables, such as food availability and predator abundance (Metcalfe *et al.*, 2002). Many of these features can vary widely from place to place and also with time, both between seasons as well as by day and night. As a consequence, fishes within their broad geographical range are rarely distributed evenly or randomly. Instead, populations typically exhibit patchy distributions, with higher abundance in preferred habitats and lower abundances (or even total absence) elsewhere (Metcalfe *et al.*, 2008). This situation becomes further complicated by the fact that individuals of most species exhibit huge changes in size as they develop from egg to adult, with the result that habitat requirements often change dramatically during their lives (Petitgas *et al.*, 2013), and it is unlikely that a single habitat will be equally suitable for all the different stages of a fish's life cycle. This feature thereby also links spatial scale to the ontogenetic component of allometric scale. Underlying these patterns of space use are environmental tolerances and thresholds that serve to limit where fish are able to thrive and survive (Fry, 1971) and directly link physiology to habitat use and movement (Huey, 1991) across life stages.

A habitat with lots of small prey and plenty of structural features, such as rocks providing refuge from larger predators, could be ideal for small, juvenile fishes, but is likely to be less suitable for larger adults of the same species that need larger prey and may be less vulnerable to predation. Given this, it might be expected that fish life histories would be characterized by spatial movement between different habitats, each of which is best for a particular activity, e.g. feeding, growing and spawning. Nevertheless, of the 25 000 species of fishes known to exist worldwide (Eschmeyer, 1998), probably only 200–300 make extensive spatial movements as adults (Harden Jones, 1980). Interestingly, migratory fish are on average more imperiled than non-migratory species and are often subject to exploitation (Donaldson *et al.*, 2011). Presumably, for most species, the costs and risks were they to perform large-scale migrations would outweigh any potential benefits (Sutherland, 1996). For other species, however, improved survival and reproductive success are achieved by moving between different habitats, and these species have therefore evolved life histories that show some ontogenetic and/or seasonal movements between habitats (e.g. Jørgensen *et al.*, 2008). Some littoral species, such as blennies (family Blenniidae), make seasonal inshore and offshore movements that extend no more than a few kilometres. In temperate waters, species such as Atlantic herring (*Clupea harengus*), mackerel (*Scomber scombrus*), Atlantic cod (*Gadus morhua*) and plaice (*Pleuronectes platessa*) make more extensive movements over several hundreds of kilometres. Finally, some species migrate over distances of several thousands of kilometres. Examples include diadromous species, such as Atlantic salmon (genus *Salmo*), Pacific salmon (genus *Oncorhynchus*) and eels (*Anguilla* species), which move between fresh water and the open sea, and the various species of tuna, billfishes and large sharks that make extensive transoceanic migrations. For some (iteroparous) species (e.g. plaice, cod and tuna), these spatial movements (migrations) are repeated annually once fish reach sexual maturity, while others undertake only a single migration to their spawning ground where, having spawned, they die (semelparous; e.g. European eels and Pacific salmon). Within this paradigm, the scales of spatial movements are very relevant to fish conservation and highly dependent on their physiology.

Physiological relevance relates to a number of factors. Most obvious is the physiological capacity for movement, in terms of both distance and rate. Some fish (e.g. eels, salmon and tuna) may cover many thousands of kilometres when moving between spawning and feeding grounds and have developed the capacities to store large amounts of energy and move very efficiently through the water. Many species have also developed the capacity for short periods of extremely rapid spatial movement that can be critical to predator avoidance and/or prey capture. The need to cover large distances may, however, not be linked entirely to swimming capability; some fish use water currents as an environmental transport mechanism. For example, the larvae of European eels (*Anguilla anguilla*) travel thousands of kilometres despite being small (<50 mm); in this, they exploit the north-easterly-moving Gulf Stream and North Atlantic Drift to transport them from the Sargasso Sea spawning area to their European freshwater biome (Baker, 1978). On a smaller scale, but involving more elaborate behaviour, plaice (*Pleuronectese platessa*) in the southern North Sea exploit tidal currents to aid their pre- and post-spawning migration (Metcalfe *et al.*, 2006), a behaviour that reduces the energetic cost of migration and leaves more resources potentially available for reproduction (Metcalfe *et al.*, 1993). The importance of allometric scaling is also evident in this example for plaice. Larvae have very limited swimming capacity in comparison to juveniles and adults but, nonetheless, can exert considerable influence on their passive transport by water currents to nursery grounds by vertically migrating and using selective tidal-stream transport (Hufnagl *et al.*, 2013). There may also be physiological requirements to tolerate altered or adverse environments, either those of the destination (particular examples are those species, such as eels and salmon, that move between marine and freshwater habitats) or those encountered *en route* between habitats (particular examples are the man-made and natural obstructions that salmon encounter in rivers when migrating from the sea to their spawning redds).

A particular challenge within conservation physiology is, therefore, to understand how to link the interactions between spatial scale as it relates to the distribution of suitable habitat and the physiological capabilities of species and life-history stages. For example, the impact of the partial loss of patchily distributed nursery areas may not arise simply as a consequence of a reduction in the total available area (i.e. a reduction in juvenile carrying capacity) but also as a consequence of an increase in the distance of open water (with high predation risk and low feeding opportunity) that has to be crossed between a spawning area and the first occurrence of suitable nursery habitat. This would likewise apply to juveniles moving between nursery areas if these become more widely separated as a consequence of habitat destruction or fragmentation. This component of ‘landscape ecology’ is becoming well recognized in terrestrial systems (Ellis *et al.*, 2012), and similar analogues and methodologies now need to be developed for marine ecosystems. Equally, scale is highly relevant to conservation in relationship to spatial management interventions, such as the establishment of marine protected areas and marine conservation zones (see “Example 1: local/regional spatial management and biotelemetry”). How big is big enough actually to have sufficient or measurable impact, and what kinds of habitats should they include? A further challenge relates to how, and to what extent, small (laboratory) and medium (mesocosm) studies can be applied to wild populations that live over large geographical areas. Which type of study is most relevant, given the trade-off between the ability to control experimental conditions and the need to understand responses to real-world situations?

**Figure 1:**
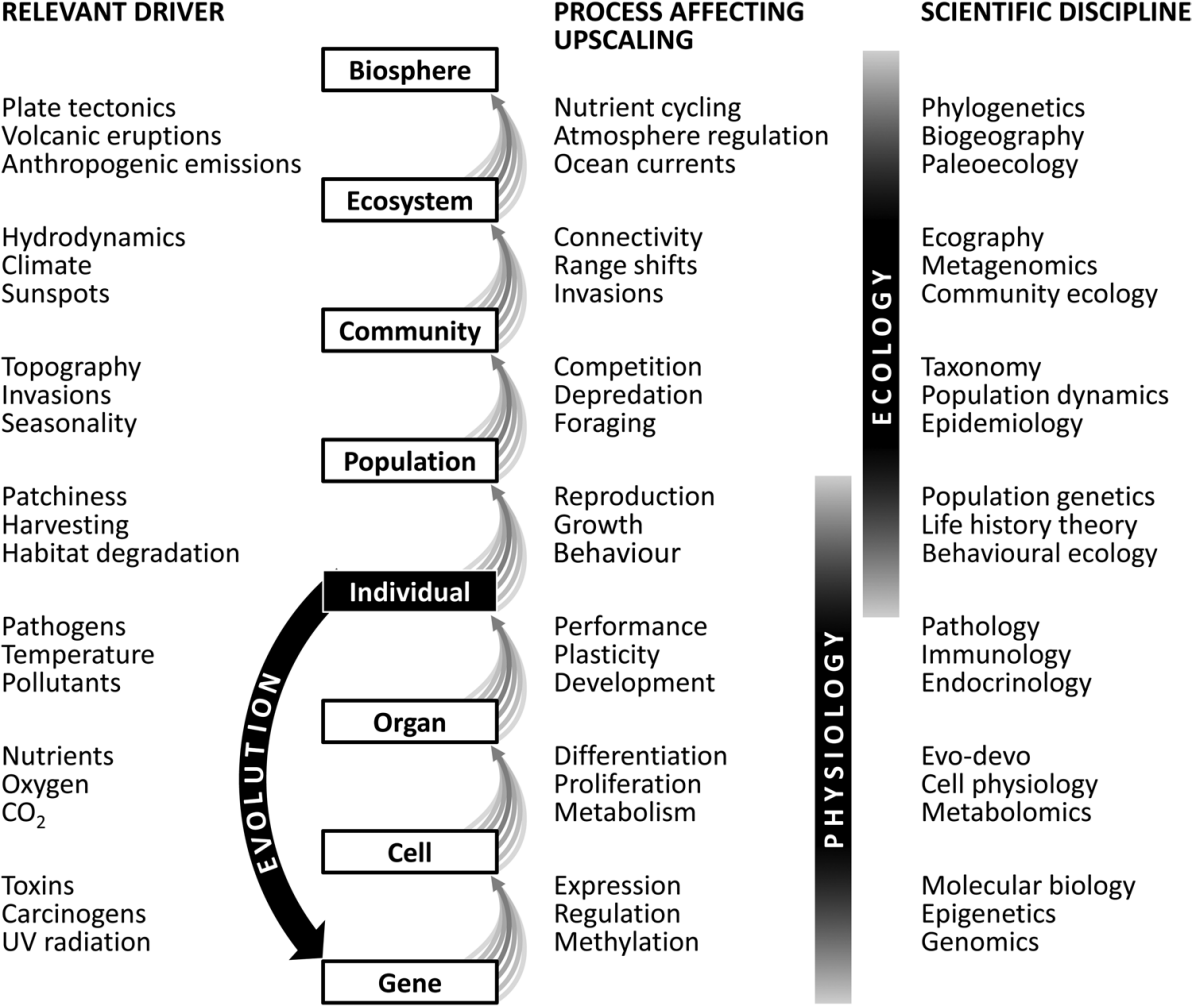
The emerging perspective of upscaling in biology. Most conservation issues relate to populations, communities or ecosystems, but it is the individual that is in contact with its local environment, and its response may often involve molecular processes within cells. Effects therefore need to be scaled up, first through molecular mechanisms to the performance of the whole organism, and then from individuals to populations and further. The bundles of grey arrows illustrate this upscaling, and how multiple entities at one level may interact to affect the next biological level. At each level, new processes may need to be taken into account, and evolution comprises an important feedback loop because Darwininan selection operating at the individual level may, over time, change the gene pool. Physiology plays a key role because its fundamental approach spans across scales from molecules to individuals and beyond. The success of conservation physiology hinges on its ability to connect with ecological disciplines that can take the scaling further, to populations, communities, ecosystems and the biosphere.

## Allometric scale

Allometry is the study of how organismal traits change with body size. Ontogenetic changes in body size are of great interest to resource managers because they are fundamental to demography and population-level processes (Young *et al.*, 2006). Many physiological, morphological, life-history and behavioural traits differ between large and small individuals and show predictable patterns of scaling as body size changes either within or among species (Schmidt-Nielsen, 1984). For many marine fishes, individuals start life only a few millimetres in length but increase in mass over several orders of magnitude to become top predators within food webs (Wieser, 1995; Killen *et al.*, 2007). For these and other reasons, any effects of body size on traits will have broad implications for fishes. For many fitness-related traits, the effects of body size are so strong that small individuals of larger species may be functionally similar (in terms of foraging requirements, for example) to large individuals of smaller species. In these cases, species distinctions may be meaningless, and some researchers have advocated a size-based approach to ecosystem analysis and stock assessment (Shin *et al.*, 2005; Jennings and Reynolds, 2007). Body size may also modify the physical manner in which organisms are affected by a given environment. For example, in an extreme case, larvae and eggs at low Reynolds numbers experience a very different set of physical challenges even when they are in the same parcel of water compared with an adult (Stanley *et al.*, 2012).

From a conservation perspective, understanding how traits scale with body size is important because certain size classes may be reproductively valuable or especially important components of food webs (Cohen *et al.*, 2003; Birkeland and Dayton, 2005) and/or more or less vulnerable to exploitation or environmental disturbance (Perry *et al.*, 2005). A worst-case scenario is when an important size class in terms of reproductive potential or community structure is also most sensitive to perturbation. In marine systems, for example, there are many fish species in which larger individuals are most fecund (Wright and Trippel, 2009) and occupy higher trophic levels but are also most vulnerable to fishing mortality (Jennings and Reynolds, 2007) or, when comparing size classes among species, have the lowest intrinsic rates of increase and potential to recover from over-exploitation (Hutchings, 2001; Denney *et al.*, 2002). Understanding how life-history traits, such as fecundity and growth rate, scale with size is critical for understanding the potential for populations to recover from over-exploitation, especially considering that fishing pressure has been shown to cause a decrease in body size within marine ecosystems (Shin *et al.*, 2005; Heath and Speirs, 2012).

There has been an especially large research focus on how metabolic rate scales with body size and, in general, smaller individuals require more energy input per unit mass compared with larger individuals (Brown *et al.*, 2004; Glazier, 2005). This relationship between metabolic demand and body mass may underlie a range of ecological phenomena and also provides a means of understanding the energetic demand of size classes and thus how energy flux among trophic levels may be altered as a result of environmental disturbance. In this manner, the allometric scaling of metabolic rate allows one to calculate how the demands on individuals translate into population-level effects that are more relevant for policy makers. Furthermore, the scaling of physiological traits, such as metabolic rate or aerobic scope, may be predictors of how animals of different sizes will respond to factors associated with climate change (sensitivity to thermal variation, hypoxia or hypercapnia; Pörtner and Farrell, 2008) or other types of environmental disturbance (e.g. rates of pollutant uptake). A major benefit stemming from accurate knowledge of allometric relationships of biological processes is that smaller animals, which are often easier to work with for logistical reasons, may be used for physiological studies in the laboratory, with results being extrapolated to larger organisms in the wild.

The most basic challenge when applying allometric relationships to conservation is establishing what those relationships actually are and the degree of precision that is required. It is common practice in fisheries models to assume a general scaling exponent (*b*) describing the slope of the log–log relationship between metabolic rate and body mass for use across all species (e.g. *b* = 0.75, a value commonly derived from data collected on endotherms; Brown *et al.*, 2004). Recent work, however, suggests not only that such exponents may be inaccurate for fishes (Post and Lee, 1996; Bokma, 2004; Killen *et al.*, 2007) but also that the precise pattern of scaling may vary among species occupying different habitats or ecological niches (Killen *et al.*, 2010; Carey *et al.* 2013). It is also possible that allometric relationships of some traits may be modulated by the environment (e.g. in response to changing temperatures, oxygenation), and so our ability to predict the effects of body size on traits may diminish in disturbed environments. The use of inappropriate scaling relationships can lead to miscalculations of energetic demands of size classes or cause inaccurate corrections for the effects of body size on physiological variables. For example, maximal metabolic rate is required for calculating aerobic scope. Despite the large body of work focusing on the scaling of standard metabolic rate in fishes, there have been only a handful of studies quantifying the scaling of maximal metabolic rate (Glazier, 2009). Although many studies use the same scaling exponent for both standard metabolic rate and maximal metabolic rate, the limited theoretical and empirical data suggest that these two variables may differ greatly in their patterns of scaling (Weibel and Hoppeler, 2005; Killen *et al.*, 2007; Glazier, 2009). This could lead to large misestimates of aerobic scope when correcting for body size and inaccurate predictions of how different size classes may cope with factors such as climate change. A final challenge when applying allometric relationships is that the effects of size may be confounded with the effects of ontogeny, making it difficult to apply trends throughout the life history of species. For example, metabolic scaling patterns during the larval or juvenile stages of many fishes appear to be different from those observed for adults (Post and Lee, 1996; Killen *et al.*, 2007).

## Phylogenetic scale

Phylogenetic scale refers to genetic relationships between species and groups of organisms shaped by evolutionary processes. It comprises macroevolution, which determines the diversity of life above the species level, often over geological time periods, by multiple processes, such as adaptive radiation and co-evolution among species. For instance, comparative hyposmotic experiments show that one killifish species, *Fundulus heteroclitus*, is able to remodel gill epithelia to cope with low salinity more rapidly than a congener (*Fundulus majalis*; Whitehead *et al.*, 2013). This result suggests higher adaptive physiological divergence along osmotic gradients within *F. heteroclitus* than *F. majalis* at the microevolutionary scale. This difference in salinity tolerance has further contributed to macroevolutionary divergence between the two species because they occupy different osmotic niches.

In comparative physiology, the phylogenetic scale has long been perceived as a nuisance because it violates the basic assumption of independence required by most statistical tests. Indeed, as early as the beginning of the 20th century, Gregory (1913) and Osborn (1917) recognized that species' variation should be partitioned between heritage (i.e. phylogenetic inertia) and adaptation. To correct for the contribution of deep-time effects when comparing trait values (e.g. temperature or hypoxia tolerance), physiologists have used methods to account for phylogenetic relatedness among species. For instance, the method of phylogenetically independent contrasts provides trait values that are statistically independent between species using phylogenetic information and a Brownian motion-like model of trait evolution (Felsenstein, 1985). The rationale is that two closely related species can have a similar physiological response to an environmental factor not only because they have a similar trait that we want to test but also because they tend to share other similar traits that are not measured. Thus, disentangling the constraints imposed by phylogeny from the influence of a particular trait on the response of organisms to an environmental stressor becomes challenging. Nowadays, evolutionary biologists have developed more fecund methods, such as the phylogenetic principal component analysis (Jombart *et al.*, 2010), that do not solely aim to remove the deep effect of evolutionary history but that take into account the relatedness of species. This new generation of approaches (Münkemüller *et al.*, 2012) should provide more complete views of multiple processes determining physiological responses of organisms.

From a biodiversity conservation perspective, phylogenetic scale has received increased attention since the seminal paper of Vane-Wright *et al.* (1991) with the objective of highlighting the need to protect not only the highest number of species but also the largest amount of evolutionary history (Mouillot *et al.*, 2011). Among the main justifications for the conservation of phylogenetic diversity (Winter *et al.*, 2013), rarity has received most of the attention because species are not equivalent within the tree of life, with some having more uniqueness or phylogenetic distinctiveness than others (Pavoine *et al*, 2005). The extinction of the former species would induce a greater loss of evolutionary history than the extinction of the latter species that share a lot of biological and genetic attributes with remaining species. Accordingly, phylogenetic rarity has entered into strategies of conservation prioritization (Redding and Mooers, 2006) but is still largely ignored for fishes in the marine realm (but see Pavoine *et al.*, 2009; Mouillot *et al.*, 2011). The next challenge would be to test whether phylogenetically rare species are also physiologically rare, with particular or unique tolerances to stressors that are hardly replaceable in the system and which can be critical in case of major disturbance events. Likewise, if conservation efforts should target sites hosting the highest levels of phylogenetic diversity (Mouillot *et al.*, 2011), one could argue that identifying and protecting physiological diversity hotspots, i.e. sites hosting fish assemblages or populations with the widest tolerances to various environmental stressors, such as in coastal lagoons and estuaries, could be a conservation priority.

Conserving the tree of life would also contribute to preserving the functional and evolutionary potential of communities. The rationale is that communities composed of weakly related species may be more productive due to complementary resource use (Cadotte *et al.*, 2008) and may cope better with environmental changes because of their diversity of responses or tolerances (Mouquet *et al.*, 2012). However, we still lack evidence that distantly related marine species have more different tolerances to stressors than do closely related species. If the level of phylogenetic conservatism is strong, it would permit inference of physiological stress responsiveness from phylogenies for many species, saving time and experiments. This would justify even further the conservation of phylogenetic diversity, as a surrogate for physiological diversity, to ensure the long-term persistence of populations and functioning of ecosystems. This should be an impetus for physiologists to perform more experiments and increase basic knowledge of stress tolerances for many species, to assess the level of physiological diversity within fish communities and so add another biodiversity component into conservation priorities.

## Integration across scales: an emerging focus on upscaling

A biological reality that is often forgotten is that it is at the level of the individual that biological processes are influenced by the environment. This does not disregard the role of large-scale environmental drivers, such as bottom trawling or climate change, where entire communities or ecosystems may be impacted simultaneously, or complex species interactions (e.g. rates of predation, facilitation, competitive dominance), but simply emphasizes that environment and response are linked at the individual and local scales. It is the sum of such impacts that traverse up the hierarchy of biological organization to have consequences for populations, communities and ecosystems. To be of relevance for conservation, a main challenge is, therefore, to scale up individual responses consistently to the level that is of relevance for societies and decision makers. Furthermore, the individual response is itself a consequence of scaling of molecular processes in single cells to whole-organism performance, or may change with body size or ontogeny and therefore involve allometric scaling.

Over the last few decades within the communities of meteorologists and oceanographers, there has been an intense focus on downscaling (Wilby and Wigley, 1997). This acknowledges three main scientific problems: (i) some drivers, such as sunspot activity and atmospheric composition, have global effects; (ii) regional climate dynamics are not independent but coupled through such factors as winds, precipitation and currents; and (iii) due to conceptual and numerical reasons, the resolution of global models will always be too coarse to resolve physical phenomena that emerge on finer spatial and temporal scales. The solution is a rigid approach whereby global models with coarse resolution feed border conditions to regional models with higher resolution, which again define the border conditions for site-specific models. There can be several such steps of downscaling upfront of a final application, both dynamic and statistical (Maraun *et al.*, 2010), and as the spatial grid size becomes smaller for each hierarchical level there is a concomitant need for shorter time steps. While global models typically use a 50 km grid size, coastal models may use a resolution as fine as 30–50 m, with the external forcing from the global model being propagated from model to model until the final resolution is achieved. Successful downscaling is good news for biology, because it is at the local scale that we need to know what the environment is, so that we can determine the consequences of an individual's environmental exposure.

Within biology, there has not been the same rigorous attention to scaling of effects as in the physical sciences, but given the disparity between the scale of measurement (individuals monitored over hours to months) and the scale of impact (populations or ecosystems over years to decades), there is an increasing need for a rigorous approach to upscaling. At most levels of the biological hierarchy, there are processes whereby the state at one level of organization affects levels higher up (Fig. 1 ).

One can argue that there are at least three important differences between biological upscaling and the downscaling used in the physical sciences. First, in scaling across the biological hierarchy of organization, there exists a natural anchor point because the environment always affects individual organisms. Environmental variability thus translates to individuals that are affected differentially, indicating at least a natural starting point at which environmental heterogeneity is going to be translated to biological variability. Second, biological species are not passively experiencing their local environment but may use behaviour actively to seek or to avoid certain environmental features, acclimatize physiologically and thus modify the response to a given environment or, more slowly, undergo source–sink dynamics across favourable and unfavourable environments, thus changing distribution over time. Third, while feedbacks are inherent also in the physical sciences (e.g. as warming reduces snow cover, which in turn increases heat absorption), evolution is a uniquely biological process, through which the scaling relationships themselves may change over time. For example, the invasion of cane toads in Australia cannot be understood by the use of constant species–environment relationships because important traits are evolving along the invasion front (Lindström *et al.*, 2013).

One can also argue that a further difference between physical downscaling and biological upscaling is the number of different scientific disciplines that are involved. Only rarely is there a single cause for a single effect in biology; more often, biology is characterized by many-to-many relationships, where several factors in the environment cause multiple concurrent responses and where a variety of traits and processes are involved. Biotic relationships are further complicated by functional diversity across species and life stages, strong feedbacks within and across species, and changing interactions due to phenotypic plasticity and evolutionary adaptation. This diversity of mechanisms is also reflected in the diversity of scientific disciplines used to study life. There are many relevant disciplines that may affect upscaling (Fig. 1), and involving them in making the right choices about which complexities to include and which to ignore will be critical for success.

Physiology plays an important role in biological upscaling because it integrates mechanisms across scales, from the molecular level through to the individual and further on to behaviour, growth and reproduction (Fig. 1). Individual-level processes are also at the heart of ecology, which extends the focus towards the population, community, ecosystem and, eventually, the biosphere as a whole. Conservation physiology specializes in understanding effects of the local environment on the individual. As most conservation issues are motivated by concerns about populations and communities, the success of conservation physiology depends on its ability to integrate its findings with ecological sciences to propagate the environmental influence all the way to the hierarchical level at which the problem needs to be resolved.

## Application of scale to policy and management

The policy realm is a complex mosaic of ‘International Law’, such as legally binding treaties; in the EU, there are ‘Directives’, ‘Statutory Instruments’ and ‘Regulations’ and, finally, there are national and regional laws enacted by individual member states as ‘Policy’ measures. The governance scale is, thus, important to consider for conservation physiologists attempting to make a meaningful contribution to inform (and potentially change) policy. Which decision maker should we influence, and is it within their power to regulate the area of our concern?

In terms of marine fishes, important international legislation includes the Convention on Biological Diversity (CBD), established in 1992, which contains general provisions that must be developed by member states. The UN Convention on the Law of the Sea (UNCLOS) in 1994 established the demarcation between national and international waters and was the driver for the establishment of the Regional Fisheries Management Organisations (RFMOs) expected to establish conservation and management measures. All member states have different authorities and policies charged with conservation and management of marine resources. Taking Europe as an example, different Regional Seas Conventions on the protection of marine environments were established, such as OSPAR, HELCOM and Barcelona (UNEP-MAP) in the early and mid 1990s for the North-East Atlantic, the Baltic Sea and offshore and coastal regions of the Mediterranean, respectively. Across all regions, the EU Common Fisheries Policy (CFP) of 1983, and recently reformed for the second time, applies. The CFP is meant to reduce the negative impacts of fisheries on the environment and will act in concert with the EU Marine Strategy Framework Directive (MSFD), which firmly establishes an ‘ecosystem-based approach’ by defining specific criteria (11 descriptors) for ‘good environmental status’. The MSFD has the explicit, regulatory objective that ‘biodiversity is maintained by 2020’, as the cornerstone for achieving good environmental status. Depending upon the European nation, specific laws or acts have been established as the instruments to carry out these EU-wide policies, which stem from broader international conventions.

Given this policy backdrop of multiple scales, how can conservation physiology best contribute? Physiologists have considerable expertise in examining cause-and-effect relationships through rigorous, controlled experimentation (Carey, 2005). This work is difficult to upscale to policy advice because conservation physiologists tend to work on individuals, whereas policies relating to the management of living marine resources are focused on populations or stocks (Cooke and O'Connor, 2010). Given both the revised CFP and the newly established MSFD, conservation physiologists working on European marine fishes will need to develop and advance tools which can not only provide advice on stocks or species but which are also relevant to the greater marine ecosystem. There is also the need to simplify the detailed, sometimes complex, physiological-based measurements into concise policy-relevant advice. Despite the differences in scale of the day-to-day activities of conservation physiologists (Cooke and O'Connor, 2010) and policy advisors (Rice, 2011), clear examples exist highlighting how information provided by the former may be immediately useful to the latter.

### Example 1: local/regional spatial management and biotelemetry

Knowledge regarding *in situ* animal movements (biotelemetry) will allow more effective spatial management measures, including the application of Marine Conservation Zones and Marine Protected Areas. Effective designs of these management tools need to take into account the scale of movement exhibited by the fish they are attempting to conserve (e.g. Parsons *et al.*, 2003; Weng, 2013). Scales of spatial movement are also relevant when considering the possible impacts of marine development. In Europe, a topical issue is offshore habitat alteration by renewable energy devices, such as wind farms and wet (wave and tide) energy converters, where both negative (barriers to migration) and positive effects on fishes (enhanced local productivity) may occur (Miller *et al.*, 2013).

### Example 2: maintenance of biodiversity and physiological optima and tolerance

Physiological knowledge is being infused within individual- and population-level models attempting to understand historical changes and project future changes in the distribution and productivity of marine fishes (Pörtner and Peck, 2010; Jørgensen *et al.*, 2012). Laboratory measurements revealing thermal windows for growth, survival and reproduction of fishes (e.g. Freitas *et al.*, 2010) are extremely useful to examine potential climate-driven changes in productivity and distribution on local (Cucco *et al.*, 2012) to global scales (Cheung *et al.*, 2011). Transferring model results or laboratory measurements to broader marine policy objectives [upscaling from individuals and populations to ecosystem-level biodiversity called for in the Convention for Biodiversity (CFB)] is challenging, but trait-based risk assessments made at the community (regional) level may offer one tractable solution (Bremner, 2008; Chown, 2012).

### Example 3: water quality criteria and interacting stressors

It is important to remember the important contribution that physiological measurements of marine fish have made in terms of aquatic toxicology and the establishment of water quality standards (Brauner *et al.*, 2007). Although much of this work has been conducted on single species having a high risk of exposure to chemical pollutants (e.g. benthic flounders and gadoids, catadromous eel or anadromous salmonids; Larsson *et al.*, 1985; Brauner *et al.*, 2007; Finn, 2007), comparison of data collected from standard tests conducted on specific life stages (particularly early life stages thought to be particularly sensitive) of various taxa are normally used to establish water quality standards. Despite the direct conduit established for information flow from physiologists (ecotoxicologists) to policy makers, debate still exists regarding how best to upscale results (from cells/tissues, to organisms to communities and ecosystems). Is a reductionist approach best when trying to establish standards protecting against ecological impacts or do emergent properties exist? How are concentrations of toxicants eliciting responses in cellular-level biomarkers, organismal-level bioassays and community-level metrics, such as functional diversity, related to one another?

## Conclusion

Our synthesis revealed that all five aspects of scale are relevant to conservation physiology, with many aspects inherently linked. We trust that this analysis will stimulate physiologists to consider how various aspects of scale represent both opportunities and challenges. Working across scales to understand mechanisms underlying conservation problems is the norm. Given that biological processes of interest (at various scales) will often not match policy and management scale, conservation physiology needs to show how it is relevant to aspects at the policy/management scale, change the scale at which policy/management intervention is applied or be prepared to be ignored. We suggest that being ignored is not an option, given that conservation physiology has both potential (see Cooke *et al.*, 2013a) and proven application (see Cooke *et al.*, 2012) to real-world conservation and resource-management problems. We have demonstrated that the concept of ‘scale’ is highly relevant to conservation physiology, particularly with respect to ensuring that such knowledge is mobilized to inform decision-making processes. Nonetheless, we agree with Cooke and O'Connor (2010) that there are some inherent challenges with respect to different scales associated with physiological research/processes and application (e.g. management, policy, decision making).

Not all ‘conservation physiology’ problems are the same. Indeed, we believe that conservation physiology can be characterized broadly into two types (Table 1). In reality, these types represent a gradient. Type A, ‘solutions for today’, represents problems that are well defined, often spatially restricted and can be addressed by several empirical studies; for example, problems associated with bycatch for a particular fishery or some form of discrete habitat alteration. Type B, ‘solutions for tomorrow’, represents more general questions or those that take decades to address, often requiring many empirical studies and a large burden of evidence before management action will occur. Issues related to climate change or the siting of marine protected areas are examples of type B issues. We suggest that conservation physiology is equally relevant to both types of issues, but that to engage practitioners and policy makers there is merit in simultaneously pursuing work that falls along the entirety of the spectrum. Being able to deliver and show successes of conservation physiology in the short term for well-defined issues is important for garnering support for longer-term research programmes. Another major conclusion arising from our analysis is that upscaling represents a potential effective strategy for making conservation physiology relevant to managers. Indeed, they may be able to ‘upscale’ type A issues to type B through creativity and ecological modelling initiatives.

**Table 1:** A conceptual framework for considering issues related to scale in conservation physiology

Scale/issue/factor	‘Type A’: solutions for today	‘Type B’: solutions for tomorrow
Specificity of question	Quite specific, e.g. a point-source disturbance/pollutant, a bycatch issue with a specific fishery	General; broad-scale environmental change phenomena where it is difficult to determine ‘who done it’
Decision makers	State/provincial/regional/sometimes national; several people, often fisheries managers, make decisions on a local level	Regional fisheries management organizations and bodies—multinational (e.g. United Nations, Committee on Fisheries, European Inland Fisheries Advisory Committee, International Council for the Exploration of the Sea)—high-level politicians
Potential for application of conservation physiology knowledge	Direct; specific studies can inform a discrete issue	Indirect; information incorporated into models and decision-support tools
Level of stakeholder engagement by researcher	Lots; including potential for citizen science, giving rapid generation of findings and ability to mobilize knowledge and act upon it	Less; not a specific stakeholder group or easy way to engage them; if stakeholders can be engaged, it is difficult to maintain interest over a long time scale
Information on which decisions are based	Potentially one or two papers/studies (may not even need to be published); may involve voluntary changes in behaviour rather than regulations or, if regulated, it is at a local scale	Burden of proof—large body of knowledge needed—likely to result in regulatory changes, but a slow process
Research time scale in terms of making significant advances towards solving a problem	Grant/thesis duration	Career(s)
Temporal scale (for making management decisions)	Short term; months to years	Long term; years to decades
Basic–applied gradient	Applied	Basic; with eventual application
Temporal scale (of biotic processes)	Days to months; often focused on scales relevant to stress and short-term mortality/behavioural impairments	Milliseconds to generations; various biotic processes
Spatial scale	Local/regional (e.g. an estuary) impacts of renewable energy and hydropower installations	National/international (e.g. the North Sea for cod and plaice, North Atlantic and Mediterranean for tuna)

It should be noted that for almost all of these issues, there is a gradient between ‘type A’ and ‘type B’ rather than two distinct categories. We submit that for conservation physiology to become a trusted source of information, it needs simultaneously to be generating success stores that result in ‘solutions for today’ (type A) and ‘solutions for tomorrow’ (type B).
